# Correction: O’Brien et al. Correction of Radiometry Data for Temperature Effect on Dark Current, with Application to Radiometers on Profiling Floats. *Sensors* 2022, *22*, 6771

**DOI:** 10.3390/s23125700

**Published:** 2023-06-19

**Authors:** Terence O’Brien, Emmanuel Boss

**Affiliations:** 1 Institute for the Study of Earth, Ocean and Space, University of New Hampshire, Durham, NH 03824, USA; 2 School of Marine Sciences, University of Maine, Orono, ME 04469, USA; emmanuel.boss@maine.edu

The authors wish to correct the following errors in the original paper [1].

The authors realized a typo in the software used for analysis in the original manuscript by which the input data, for both *E_d_* and iPAR, were scaled by a factor of 100. Therefore, the reported coefficients and corrections were all a factor of 100 higher for the reported units. This erroneous scaling factor was applied to all wavebands and iPAR in both data used to compute the sensor-specific, temperature-dependent dark corrections (Equation (1) in the manuscript) and in the profile data to which the corrections were applied (by Equation (5) in the manuscript). We make the following corrections.

## 1. Text Correction

The following correction has been made to the Abstract:

The corrections are based on modeling the temperature of the radiometer and show an average bias in the measured value of nearly $1\times 10^{-4}$ ${W\;m}^{-2}{\;\mathrm{nm}}^{-1}$, an order of magnitude larger than the reported uncertainty of $2.5\times 10^{-5}$ ${W\;m}^{-2}{\;\mathrm{nm}}^{-1}$ for the sensors deployed on BGC-Argo floats (SeaBird scientific OCR504 radiometers).

In the subsection *Profile Extraction, Quality Control and Modeling* under Section 2: Materials and Methods, in the sixth paragraph, we make the correction:

We constrained measurements to the range |$E_{d}$| < $3\times 10^{-4}$ ${W\;m}^{-2}{\;\mathrm{nm}}^{-1}$ and |*PAR*| < 0.5 ${\mu \mathrm{mol}\;\mathrm{photons}\;m}^{-2}\;s^{-1}$.

In the subsection *Profile Extraction, Quality Control and Modeling* under Section 2: Materials and Methods, in the seventh paragraph, we make the correction:

This test is important as *dEd/dTs* is small ($-3\times 10^{-5}$ to $3\times 10^{-5}$ W m^−2^ nm^−1^ °C^−1^) and hence not detectable relative to other environmental processes if the temperature gradient in a profile is too small.

In Section 3: Results, in the second paragraph, we make the correction:

$x_{1}$ ranges from $-3.4\times 10^{-5}$ to $2.3\times 10^{-5}$ (${W\;m}^{-2}{\;\mathrm{nm}}^{-1}\;{}^{\circ}C^{-1})$ by method 2 compared to $-2.4\times 10^{-5}$ to $1.2\times 10^{-5}$ (${W\;m}^{-2}{\;\mathrm{nm}}^{-1}\;{}^{\circ}C^{-1})$ by method 1 (Figure 1).

In Section 4: Discussion and Summary, in the first paragraph, we make the following correction:

For this BGC-Argo dataset, the mean absolute temperature corrections on $E_{d}\;$using night and day profiles are $8\times 10^{-5}\;$and $9.3\times 10^{-5}$ $\left({W\;m}^{-2}{\;\mathrm{nm}}^{-1}\right)$ and maximum absolute corrections are $4.4\times 10^{-4}$ and $6.14\times 10^{-4}$ $\left({W\;m}^{-2}{\;\mathrm{nm}}^{-1}\right)$, respectively (Table 2). These corrections are more than an order of magnitude larger than the known sensitivity of the sensors ($2.5\times 10^{-5}$ ${W\;m}^{-2}{\;\mathrm{nm}}^{-1})$.

In Section 4: Discussion and Summary, in the fourth paragraph, we make the following correction:

They provide a model that includes drift correction, where in certain, but rare, cases they observed a drift as much as $1\times 10^{-7}\;days^{-1}$, producing a significant correction over a three-year lifetime. 

In Section 4: Discussion and Summary, in the fourth paragraph, we make the following correction:

The values of our modeled coefficients $dE_{dark}/dT$ agree well with [4] and [5], with the maximum $dE_{dark}/dT$ on the order of $2-4\times 10^{-5}$ ${W\;m}^{-2}{\;\mathrm{nm}}^{-1}{℃}^{-1}$ (Figure S9 in [5]). 

In Section 4: Discussion and Summary, in the fourth paragraph, we make the following correction:

For $dPAR_{dark}/dT$, ref. [4] produces the smallest values, on the order of $2\times 10^{-2}$ ${\mu \mathrm{mol}\;\mathrm{photons}\;m}^{-2}\;s^{-1\;}{℃}^{-1}$, while [5] agrees with our maximums as high as $4\times 10^{-2}$ $\mu {\mathrm{mol}\;\mathrm{photons}\;m}^{-2}\;s^{-1\;}{℃}^{-1}$. Likewise, we find a similar model constant of PAR (our $x_{0})$ with [5] showing the highest maximum. Our investigation of the daytime profiles revealed these significant dark readings at depth, and our corrections for PAR are of the same order relative to surface values as our corrections for *E_d_*: at 10 m, our average PAR correction is on the order of 0.001% of the 10 m measured PAR value, analogous to the average 10 m correction at all three wavelengths. Overall we find very similar results between our method and [4,5]. 

## 2. Error in Table

We update Table 1, Table 2 and Table 3 from the original publication to adjust all values by the scaling factor of 100 by adjusting the presented values to the reported units. The corrected tables appear below:

## 3. Error in Figure

We update Figure 1, Figure 2, Figure 3, Figure 4, Figure 5 and Figure 6 from the original publication to adjust all values by the scaling factor of 100 by adjusting the presented values to the reported units. The corrected figures appear below. 

We apologize for the inconvenience or confusion caused by our error in the original manuscript. The changes do not affect the method presented, but they do affect the magnitude of the bias identified in the sensors. The authors state that the scientific conclusions are unaffected. This correction was approved by the Academic Editor. The original publication has also been updated.

## Figures and Tables

**Figure 1:**
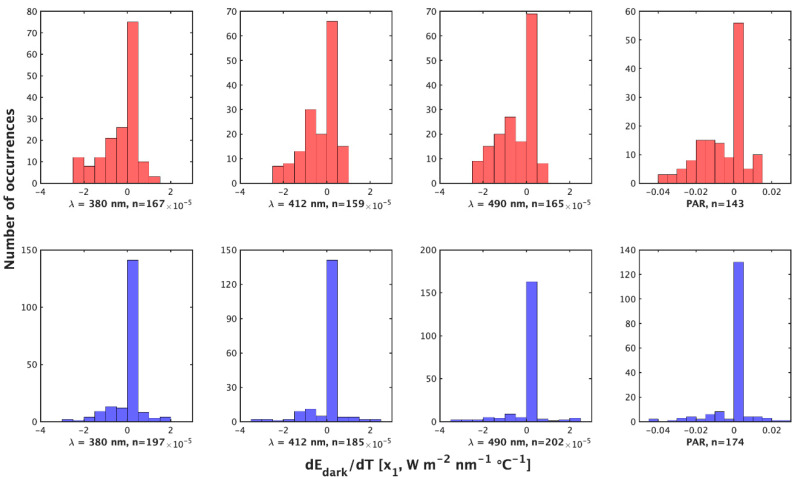
Histograms of the value of $x_{1}$ = $dE_{d}/dT_{s}$ (${W\;m}^{-2}{\;\mathrm{nm}}^{-1}\;{}^{\circ}C^{-1}$) by the night method (**top**, red) and day method (**bottom**, blue) for λ = 380 nm, 412 nm, 490 nm, and PAR (**left** to **right**).

**Figure 2:**
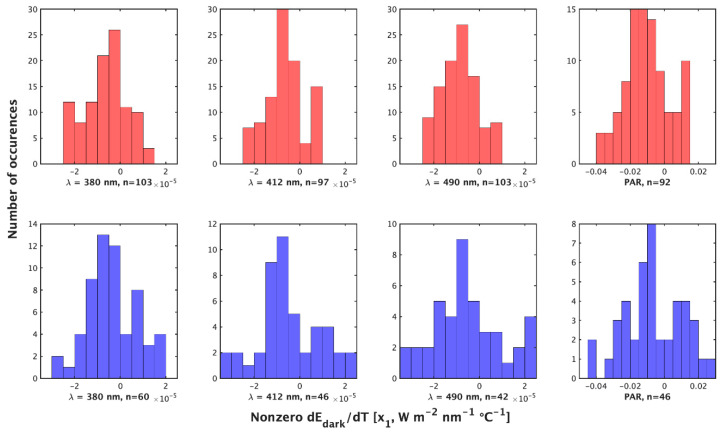
Histograms of the non-zero value of $x_{1}$ = $dE_{d}/dT_{s}$ $\left({W\;m}^{-2}{\;\mathrm{nm}}^{-1\;}{℃}^{-1}\right)$ or $dPAR/dT_{s}$ (${\mu \mathrm{mol}\;\mathrm{photons}\;m}^{-2}\;s^{-1\;}{℃}^{-1}$) by the night method (**top**) and day method (**bottom**) for (**left** to **right**). λ = 380 nm, 412 nm, 490 nm, and PAR.

**Figure 3:**
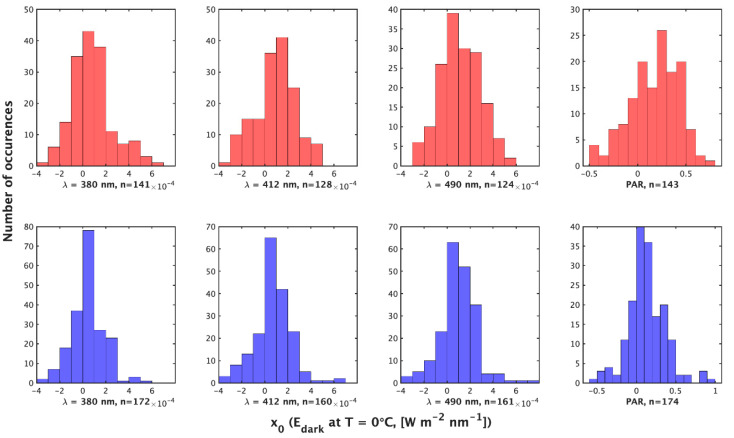
Histograms of the value of $x_{0}$ (${W\;m}^{-2}{\;\mathrm{nm}}^{-1\;}$ or ${\mu \mathrm{mol}\;\mathrm{photons}\;m}^{-2}\;s^{-1}$) by the night method (**top**) and day method (**bottom**) for (**left** to **right**) λ = 380 nm, 412 nm, 490 nm, and PAR. $x_{0}$ is the value reported by the irradiance sensor in the dark at $T_{s}=0\;℃$.

**Figure 4:**
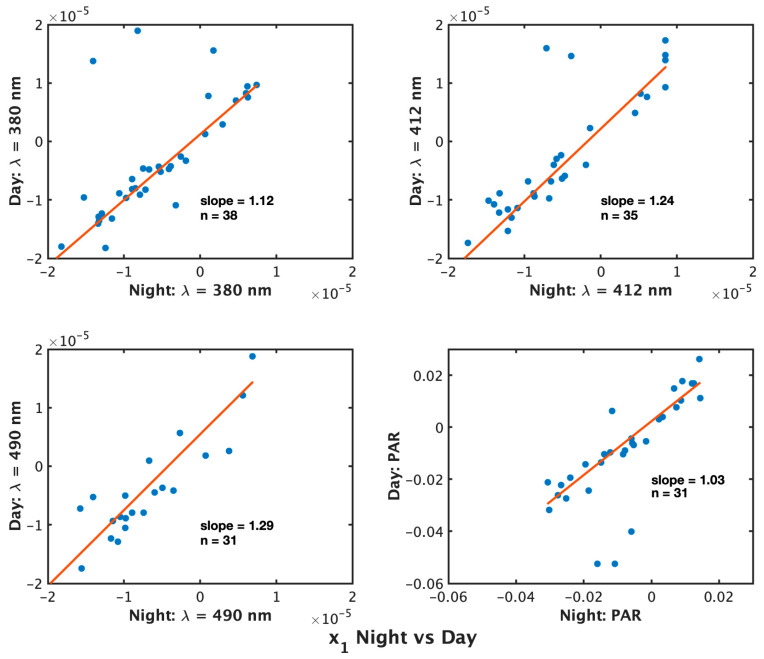
Comparison of $x_{1}$ obtained from nighttime profiles (x-axis) and daytime profiles (y-axis) $({W\;m}^{-2}{\;\mathrm{nm}}^{-1\;}{℃}^{-1}$ or ${\mu \mathrm{mol}\;\mathrm{photons}\;m}^{-2}\;s^{-1\;}{℃}^{-1}$) for floats that produced non-zero $x_{1}$ using both methods. Results are presented for λ = 380 nm (**top left**), 412 nm (**top right**), 490 nm (**bottom left**), and PAR (**bottom right**).

**Figure 5:**
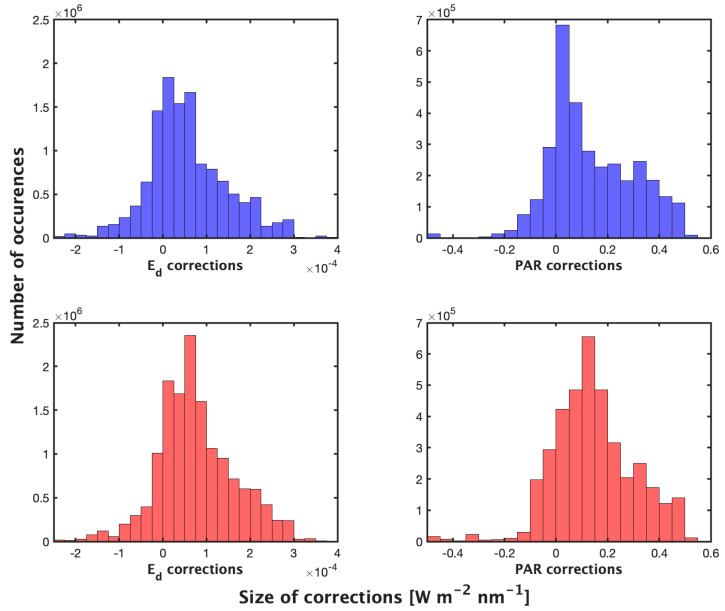
Size of corrections applied by the night (**top**) and day (**bottom**) method on good profiles at all wavelengths (19,605,908 measurements corrected) (${W\;m}^{-2}{\;\mathrm{nm}}^{-1\;}$ or ${\mu \mathrm{mol}\;\mathrm{photons}\;m}^{-2}\;s^{-1}$). Statistics shown in Table 2.

**Figure 6:**
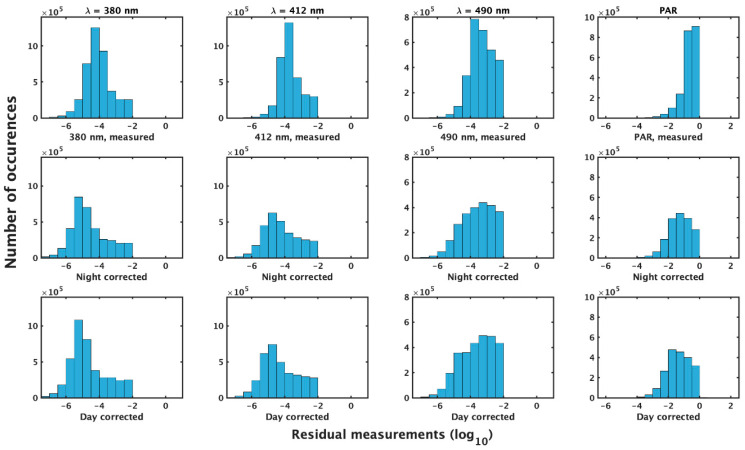
Measurements of $E_{d}$ (λ, z) < 0.01 ${W\;m}^{-2}{\;\mathrm{nm}}^{-1\;}$and PAR(z) < 1 ${\mu \mathrm{mol}\;\mathrm{photons}\;m}^{-2}\;s^{-1\;}$ (**top** row) after corrections are applied by the night (**middle** row) and day (**bottom** row) methods. Columns are (**left** to **right**) λ = 380, 412, 490 nm, and PAR. Plotted on $log_{10}$ scale.

**Table 1:** Nonzero $x_{1}$ by the night method and day method for all λ (${W\;m}^{-2}{\;\mathrm{nm}}^{-1\;}{℃}^{-1}{\;\mathrm{or}\;\mu \mathrm{mol}\;\mathrm{photons}\;m}^{-2}\;s^{-1\;}{℃}^{-1}$), as shown in Figure 2.

Method	Median	IQR	Mean	SD
Night *E_d_*	$-6.7\times 10^{-6}$	$1\times 10^{-5}$	$-7\times 10^{-6}$	$8.5\times 10^{-6}$
Day *E_d_*	$-5.8\times 10^{-6}$	$1.73\times 10^{-5}$	$-4.4\times 10^{-6}$	$1.3\times 10^{-5}$
Night PAR	$-1.064\times 10^{-2}$	$1.674\times 10^{-2}$	$-9.7\times 10^{-3}$	$1.31\times 10^{-2}$
Day PAR	$-9.53\times 10^{-3}$	$2.858\times 10^{-2}$	$9.6\times 10^{-3}$	$1.98\times 10^{-2}$

**Table 2:** Absolute size of corrections applied by both methods on good profiles at all wavelengths and PAR (19,605,908 measurements corrected) (${W\;m}^{-2}{\;\mathrm{nm}}^{-1\;}{\;\mathrm{or}\;\mu \mathrm{mol}\;\mathrm{photons}\;m}^{-2}\;s^{-1}$).

Method	Max	Median	IQR	Mean	SD
Night *E_d_*	$4.44\times 10^{-4}$	$5.7\times 10^{-5}$	$9.3\times 10^{-5}$	$8.0\times 10^{-5}$	$7.2\times 10^{-5}$
Day *E_d_*	$6.14\times 10^{-4}$	$7.1\times 10^{-5}$	$1.01\times 10^{-4}$	$9.3\times 10^{-5}$	$7.4\times 10^{-5}$
Night PAR	$5.52\times 10^{-1}$	$1.09\times 10^{-1}$	$2.3\times 10^{-1}$	$1.6\times 10^{-1}$	$1.41\times 10^{-1}$
Day PAR	$6.86\times 10^{-1}$	$1.4\times 10^{-1}$	$1.8\times 10^{-1}$	$1.72\times 10^{-1}$	$1.31\times 10^{-1}$

**Table 3:** Measurements for all λ of measured $E_{d}$ (λ, z) < 0.01 ${W\;m}^{-2}{\;\mathrm{nm}}^{-1}$ and PAR(z) < 1 ${\mu \mathrm{mol}\;\mathrm{photons}\;m}^{-2}\;s^{-1}$, corrected by the night and day methods (*n* = 12,930,490), as shown in Figure 6.

Method	Median	IQR	Mean	SD
Measured *E_d_*	$1.66\times 10^{-4}$	$5.76\times 10^{-4}$	$9.05\times 10^{-4}$	$1.82\times 10^{-3}$
Night corrected *E_d_*	$4.8\times 10^{-4}$	$5.12\times 10^{-4}$	$8.2\times 10^{-4}$	$1.82\times 10^{-3}$
Day corrected *E_d_*	$3.5\times 10^{-4}$	$5.05\times 10^{-4}$	$8.2\times 10^{-4}$	$1.83\times 10^{-3}$
Measured PAR	$2.65\times 10^{-1}$	$3.24\times 10^{-1}$	$3.18\times 10^{-1}$	$2.48\times 10^{-1}$
Night corrected PAR	$5.03\times 10^{-2}$	$1.77\times 10^{-1}$	$1.37\times 10^{-1}$	$2.09\times 10^{-1}$
Day corrected PAR	$3.9\times 10^{-2}$	$1.78\times 10^{-1}$	$1.13\times 10^{-1}$	$2.09\times 10^{-1}$
